# A Rare Case of Intraoral Psoriasis

**DOI:** 10.7759/cureus.5204

**Published:** 2019-07-22

**Authors:** Divyambika C Venugopal, Sathasivasubramanian S, Malathi Narasimhan

**Affiliations:** 1 Oral Medicine and Radiology, Faculty of Dental Sciences, Sri Ramachandra Institute of Higher Education and Research, Chennai, IND; 2 Oral Pathology, Faculty of Dental Sciences, Sri Ramachandra Institute of Higher Education and Research, Chennai, IND

**Keywords:** psoriasis, mucocutaneous, papulosquamous

## Abstract

Psoriasis is a chronic inflammatory mucocutaneous disease predominantly affecting the skin. While oral manifestations are common in many papulosquamous conditions, the occurrence of oral lesions in psoriasis is relatively rare. This case report highlights the presence of oral lesions in cutaneous psoriasis and the need for early identification of such lesions for timely management and better symptomatic improvement.

## Introduction

Psoriasis is a commonly documented skin disorder with a clinical presentation of papules and plaques covered with white scales. However, the intraoral presentation of psoriasis, either coexisting with cutaneous lesions or presenting as isolated oral lesions, is rarely reported in the literature [1]. Among the various clinical presentations involving the skin, the plaque type is more common, followed by guttate, pustular, inverse, and erythrodermic types [2]. The most commonly reported oral manifestations are erythema migrans, gingival erythema, psoriatic arthritis of the temporomandibular joint, and, very rarely, psoriatic mucositis affecting buccal mucosa and palate [3,4]. A review by Brooks shows evidence of an increased risk for an array of systemic disorders in the presence of cutaneous psoriasis and emphasizes the need for a thorough evaluation of systemic comorbidities for successful clinical outcomes [3].

## Case presentation

**Figure 1:**
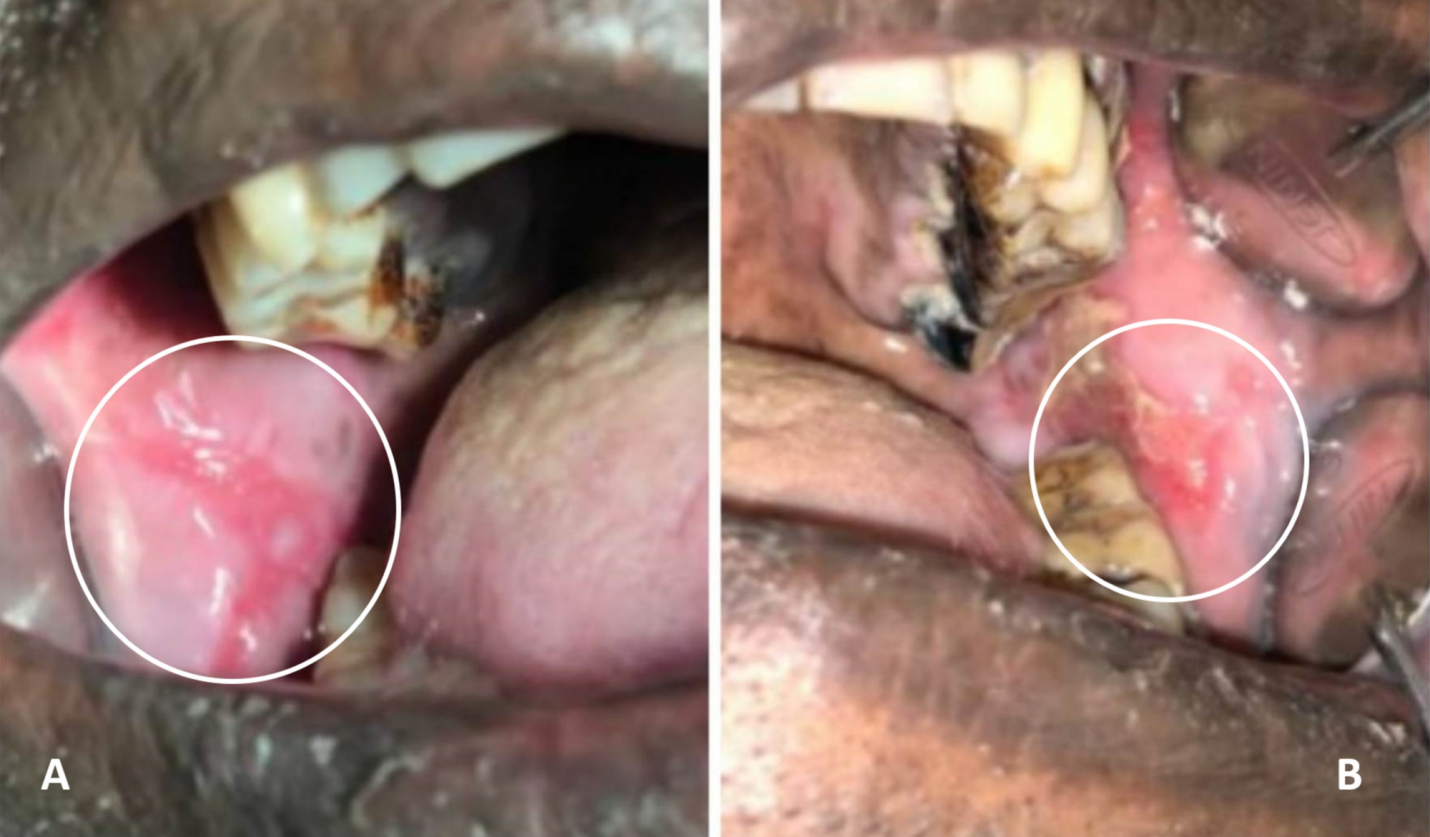
Intra-oral lesion on the right (A) and left (B) buccal mucosa

A 56-year-old male patient reported to the Department of Oral Medicine and Radiology, with a chief concern of an oral burning sensation on consuming spicy foods lasting three weeks. The patient’s history was significant for cutaneous psoriasis on the right nape of the neck and nail beds, being treated with medication. He reported symptomatic improvement with respect to cutaneous psoriasis following treatment. Intraoral examination revealed well-defined linear whitish plaque over the right and left buccal mucosa, surrounded by erythematous areas and multiple erythematous lesions in relation to the maxillary and mandibular labial mucosa. The whitish plaque was non-scrapable and non-tender (Figure 1) 

**Figure 2:**
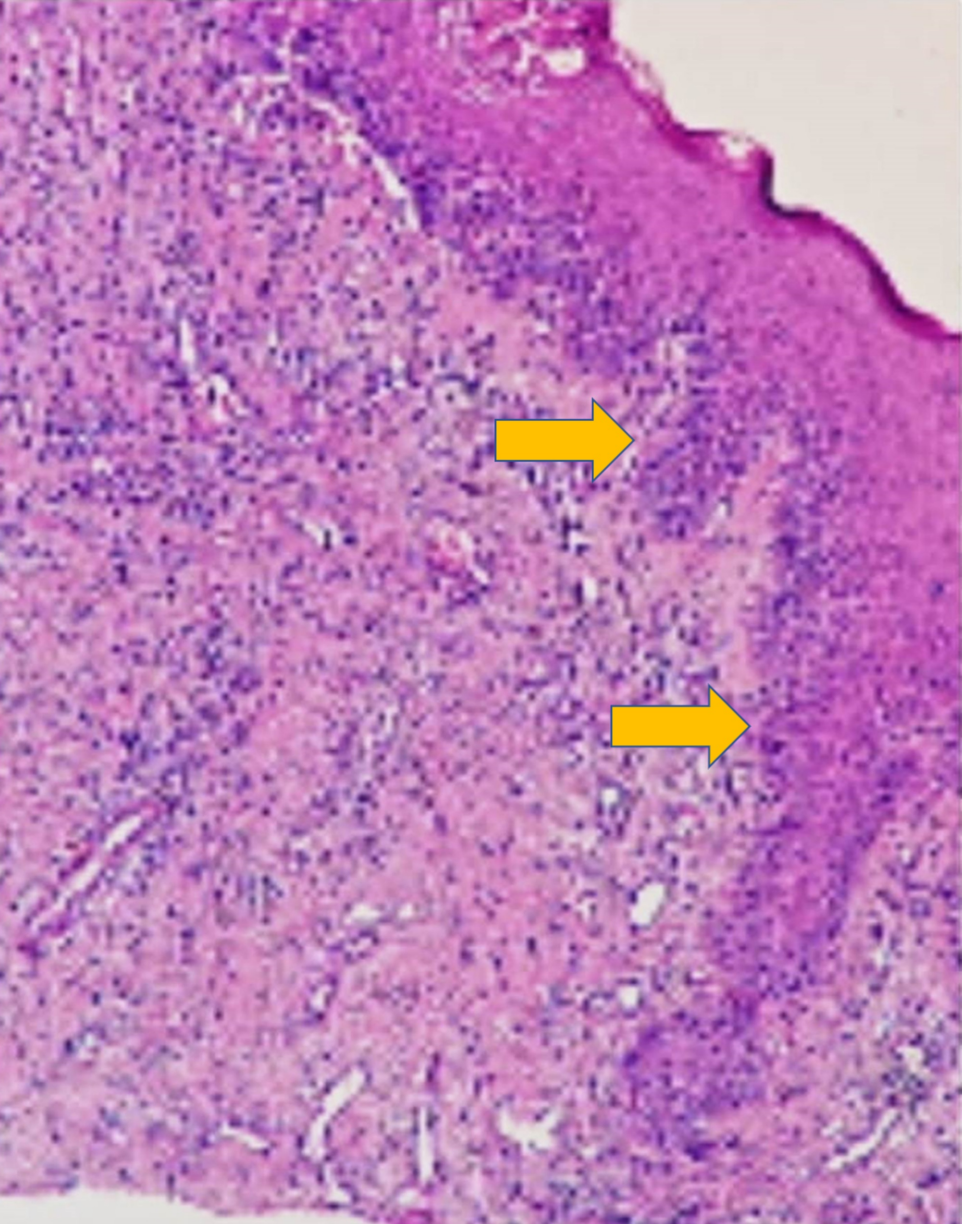
10X view showing parakeratotic stratified squamous epithelium with test-tube shaped rete ridges

**Figure 3:**
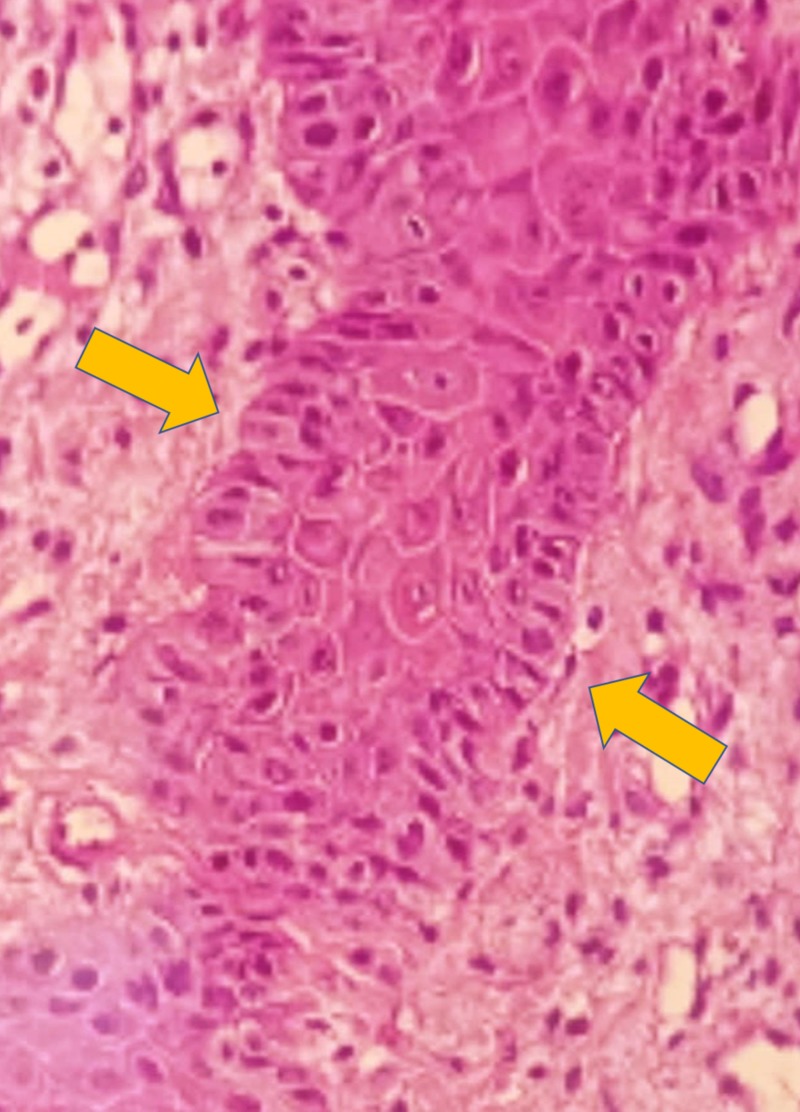
40X view showing liquefactive basal cell degeneration with test-tube shaped rete ridges

The results of the patient’s hematological investigations were unremarkable. An incisional biopsy was performed, and the histopathological examination revealed the presence of parakeratotic stratified squamous epithelium with acanthosis alternating with atrophic epithelium with a focal area of liquefactive degeneration of basal test tube-shaped rete ridges (Figures 2, 3)

**Figure 4:**
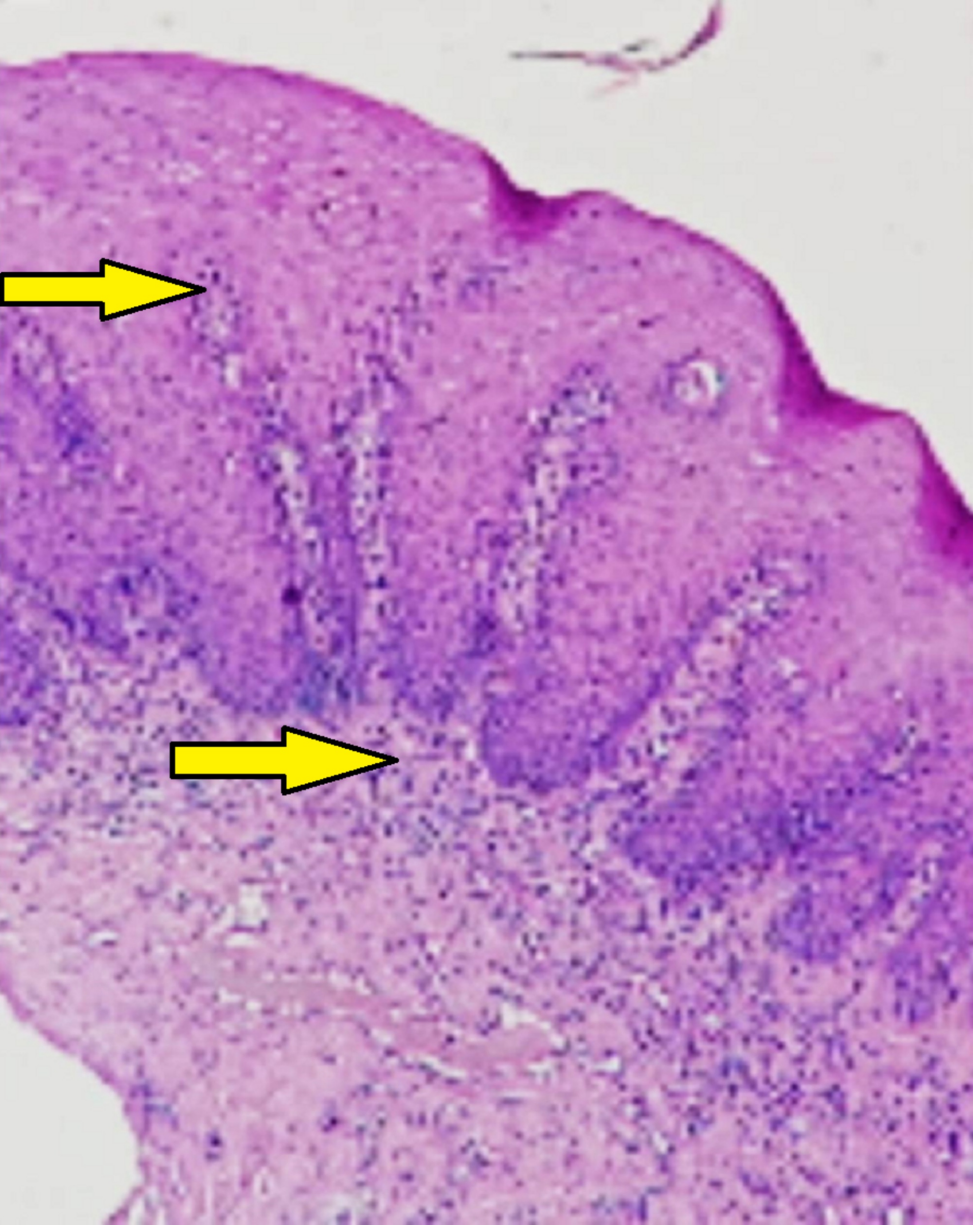
10X view showing papillomatous hyperplasia with juxta epithelial inflammatory cell infiltration

**Figure 5:**
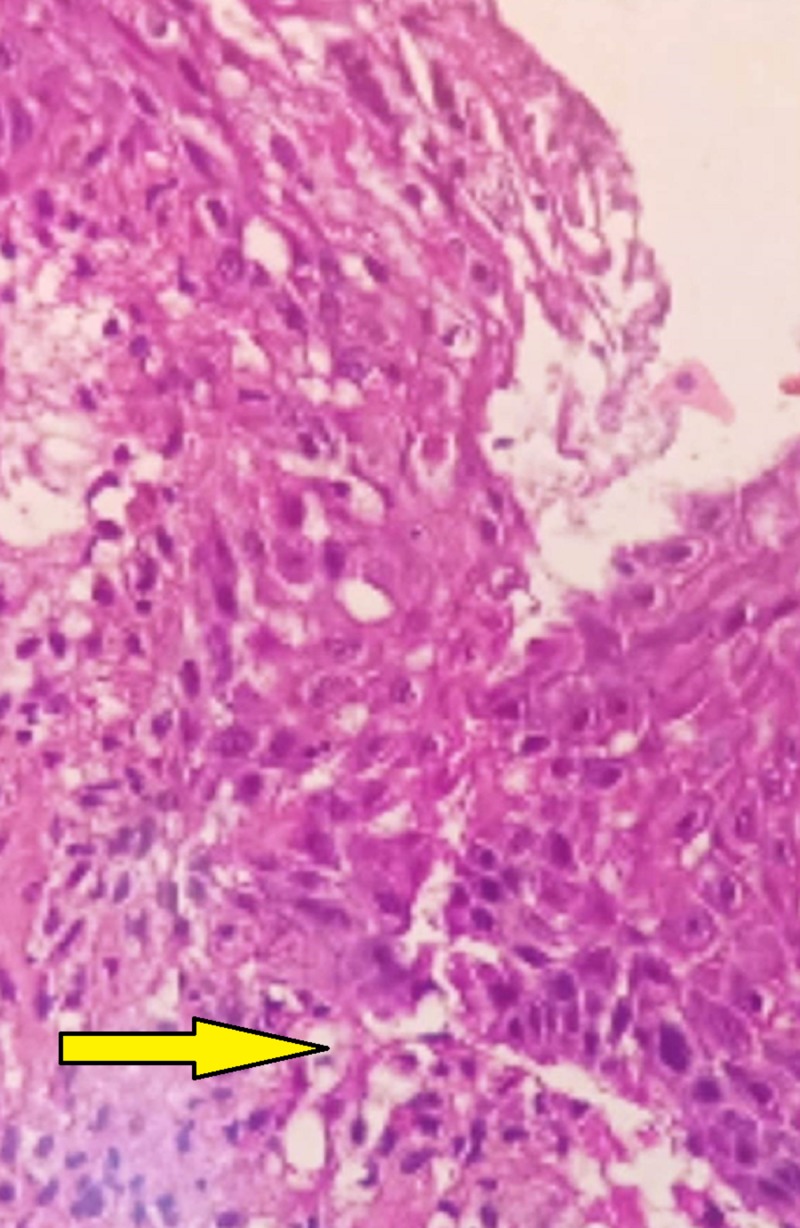
40X view showing Munro’s abscess

The underlying connective tissue showed focal areas of juxta epithelial hyalinization along with chronic inflammatory cell infiltration and increased vascularity (Figures 4, 5).

The patient was advised topical steroids (triamcinolone acetonide 0.1%) twice per day for 15 days. He was relieved of the burning sensation but was ultimately lost to follow-up.

## Discussion

Psoriasis is a chronic inflammatory disorder, predominantly affecting the skin, and has been one of the oldest documented skin lesions [5]. Oral manifestations of psoriasis are relatively rare, which may be due to the more rapid epithelial regeneration in the oral cavity as compared to the skin [5]. The first case of oral psoriasis was reported by Oppenheim in 1903, where the patient had presented with well-demarcated yellowish-white plaques, subsequently confirmed by biopsy [6]. Some of the oral manifestations previously reported include geographic stomatitis and fissured tongue (occurring in 10% of patients with skin manifestations) [5].

Unlike skin lesions, oral lesions do not have a regular pattern and varied presentations include small, whitish papules that produce bleeding points on scraping; red and white plaques that mimic dermal presentation; and bright red or white patches ranging in severity, sometimes with a simultaneous presentation of angular cheilitis [7,8]. The present case had a bilateral presentation of non-scrapable atypical lesions and white plaque surrounded by erythematous areas with multiple erythematous lesions in the maxillary and mandibular labial mucosa, which did not fit into similar recognized white lesions. Some guidelines indicate that oral psoriasis diagnosis should only be made in the presence of concomitant skin lesions [5]. However, isolated cases of oral psoriasis have been documented [9,10]. Our patient was undergoing treatment for guttate psoriasis of the skin. Hence, correlating skin lesions with histological findings from oral biopsy favored the final diagnosis of oral psoriasis. Treatment usually aims for symptomatic improvement [5]. The mainstay of treatment includes topical and systemic steroids, immunomodulators such as cyclosporine and tacrolimus. Patients have shown symptomatic improvement and regression of oral lesions following topical application of topical corticosteroids [7,9]. As per the US guidelines on the management of psoriasis, topical corticosteroids are commonly used in the management of psoriasis either as a single agent therapy in case of mild to moderate disease or in combination with systemic steroids in severe disease to maximize control and minimize side effects [11]. The topical corticosteroids are commonly used in painful oral inflammatory conditions such as erosive lesions of the oral mucosa [12]. Oral psoriasis has been successfully managed with topical steroids with complete remission [9,10]. Despite being on a low dose of systemic steroids, our patient experienced symptomatic improvement with a reduction in the burning sensation and regression of oral lesions only after topical application of triamcinolone acetonide, which highlights the role of topical steroids in successful management of such erosive conditions.

## Conclusions

This case highlights an atypical presentation of oral psoriasis coexisting with cutaneous psoriasis. Obtaining an accurate medical history along with a thorough evaluation aids in the early identification of the condition, thus facilitating the prompt institution of treatment for better clinical outcomes.
